# Social media use as a coping mechanism during the COVID-19 pandemic: A multidimensional perspective on adolescents' well-being

**DOI:** 10.3389/fpubh.2022.1062688

**Published:** 2023-01-11

**Authors:** Alexandra Maftei, Ioan-Alex Merlici, Oana Dănilă

**Affiliations:** Faculty of Psychology and Education Sciences, Alexandru Ioan Cuza University, Iaşi, Romania

**Keywords:** adolescents, social media, engagement, emotions, well-being, depression

## Abstract

**Introduction:**

Social media use was previously characterized as both a maladaptive coping mechanism, and a source of engagement with peers, suggesting an ambivalent effect. The present study explored how adolescents might use social media as a coping mechanism during the COVID-19 pandemic, using a multidimensional perspective on well-being.

**Methods:**

Our sample consisted of 259 Romanian teenagers aged 11–16 (*M* = 13.38, *SD* = 0.93, 57% males). We investigated the potential indirect effect of social media use, i.e., its cognitive, affective, and behavioral dimensions on the relationship between depressive symptoms and adolescents' well-being.

**Results:**

Across all mediation analyses, our results suggested that social media use positively predicted adolescents' well-being. Given the multidimensional approach to both social media use and well-being, our findings suggested that adolescents' well-being was predicted not only by actual social media use behaviors but also by cognitions related to the expectation of receiving gratification on social media and the intense affective states related to the desire to use social media. Also, our data suggested that adolescents with high levels of depressive symptoms might be more likely to capitalize on social media use and have expectations related to receiving approval from others in the context of social media use.

**Discussion:**

Depressive symptoms might be more relevant when explaining the cognitive and affective involvement during social media use. However, their ability to predict the actual social media use behaviors may be limited. Furthermore, adolescents that present depressive symptoms might be more prone to use social media, in order to improve their well-being.

## Introduction

Adolescence is a fascinating and challenging period of self-discovery. From a biological point of view, the beginning of puberty can signal the beginning of adolescence (1). Socially, however, adolescence is generally characterized by growing independence from parents, the increased influence of the peer group, and other aspects such as frequent mood swings, the impact of people from the same age group, the need to build an identity, and the fear of social rejection, which all play an essential role in determining behaviors, emotional reactions, and the formation of coping strategies (1, 2). In addition, a more systemic perspective also underlines the significant role of individual and cultural variability (i.e., more significant than age-related norms) that might influence the developmental tasks achieved during this stage [e.g., (2)].

Among the challenges of adolescence, the development of a secure and stable sense of identity is critical since it can contribute to the shaping of sexuality (both in terms of identity and orientation) (3), the development of intimacy in various types of relations, strengthening the autonomy and different achievements, particularly associated with the educational path (4). Moreover, the COVID-19 pandemic has brought substantial challenges to people of all ages, youngsters included, imposing unprecedented, unpredictable changes.

In this context, previous studies highlighted the hardship experienced by adolescents and their caregivers (5), with a particular increase in experienced difficulties due to the COVID-19 pandemic (6, 7). At the same time, a growing body of research also emphasized teenagers' variety of coping resources and personal strengths (8), with technology playing a significant role in this regard (9). Building on this perspective, the present study aimed to explore how social media might be used as a coping mechanism during the COVID-19 pandemic, using a multidimensional perspective on adolescents' well-being.

## Adolescents' well-being: The EPOCH model

Positive psychology devoted increased attention in the last decades to happiness, well-being, or life satisfaction, all of them describing a common feature, i.e., the constant and long-lasting presence of positive feelings, emotions, and outcomes for a person (10). Some positive psychologists reject hedonistic theories in favor of Aristotelian or eudaemonic views of well-being (11). For the conceptualization of well-being in the present study, we used the EPOCH paradigm, which understands well-being as a variable structured on five dimensions: *engagement* in activities, *perseverance, optimism, connection* with the people around us, and *happiness* (12).

### Engagement

The EPOCH model's engagement dimension refers to adolescents' active and voluntary participation in activities in different areas of life (social, professional, and educational) (13). Engagement implies a strong motivation for adolescents to pursue their goals and passions and to take the initiative to start enjoyable or exciting activities. In other words, engagement refers to adolescents' ability to become absorbed in what they do (with its most intense form referring to a sense of flow), a state of complete absorption with the loss of a sense of time and self (14). Recent studies (15) suggested a significant link between engagement and educational mastery goals.

### Perseverance

Within the EPOCH model, perseverance is conceptualized as an adolescent's ability to accomplish personal goals despite encountering obstacles (12). Previous studies suggested significant associations of persistence in educational contexts with the establishment of harmonious relationships. For example, in the study by Tian et al. (16), which involved 1,476 adolescents, social support in the educational context, both from teachers and colleagues, had significant associations with the participants' subjective well-being. Also, some studies have suggested a bidirectional relationship between persistence and the other dimensions of well-being and adolescent school performance (17).

### Optimism

The EPOCH model's optimism dimension refers to adolescents' orientation toward self-confidence, hope, adopting positive attitudes related to the future, and anticipating positive long-term results (12). Furthermore, Zeng et al. (18) suggested that optimism is a central feature of adolescent well-being, and similar results were reported in subsequent studies. For example, Zou et al. (19) suggested that optimism seems to be a significant predictor of life satisfaction among adolescents.

### Social connectedness

Within the EPOCH model, social connectedness refers to establishing and maintaining harmonious social relationships with family members and relevant others. Furthermore, these relationships are bidirectional, with adolescents' perceptions of their relationship with other people and those people's perceptions of adolescents being important in establishing social connections. Previous studies suggested social connectedness as a central dimension in determining adolescent well-being and that the feelings of belonging and integration in the school social environment mediated the relationship between students' academic and social skills and dimensions of social connection, happiness, and optimism within well-being (20, 21). Furthermore, other studies [e.g., (22)] suggested that adolescents' low social connectedness within middle school social groups is positively associated with depressive and anxiety symptoms.

### Happiness

Of the five dimensions of the EPOCH approach, happiness is a particularly controversial one, as the scientific community is divided between adherents of positivist psychology, which positions happiness as a central concept in the assessment of well-being, and those who oppose this approach, considering happiness as a subjective construct and difficult to measure and assess (23). However, the EPOCH approach integrates the concept of happiness alongside other dimensions into the central idea of well-being. The results presented by Lukoševičiute et al. (24), in which 133 studies on the subjective happiness of adolescents were included, suggested that most studies [i.e., 64] used a single item to measure subjective happiness, and only 18 of them had validation procedures. However, adolescent happiness seems to be positively associated with social connectedness and optimism (21), highlighting its relevance for the optimal development of adolescents' well-being.

## Adolescents' social media use

Addressing any issue concerning adolescence today without considering social media and its presence would mean ignoring a significant factor. The “digital natives” concept refers to the generations that have had access to digital technologies (e.g., computers, smartphones) since childhood (25, 26). The growing literature concerning the features of the digital natives has also highlighted the existence of the Z generation (children born between 1995 and 2010) (27) and the alfa generation (born 2010+) (28).

Digital natives might be different from children and teenagers of other generations due to their early and continuous exposure to a much faster and broader flow of information, studying, writing, and interacting with each other in ways that are very different from previous generations (29). As such, the rise of new social interaction methods (such as social media networks) is also strongly connected with the characteristics and behaviors of digital natives (30).

The Internet makes available, both to teenagers and people of other age groups, a series of tools that simplify various social and cognitive processes (31) and tasks that would require, in its absence, an increased effort. Although early digitally exposed teenagers (as the ones in the present study) have access to vast sources of information, new methods of socialization and entertainment from a very young age, the potential positive or negative effects of this factor are still a debated subject in the scientific community (32). Among adolescents' negative aspects of Internet access, disrupting the circadian rhythm and reducing sleep hours are expected consequences of excessive Internet use (33). Furthermore, Internet, smartphones, and video game addictions are also highly prevalent among teenagers, with higher rates among male adolescents (34). More importantly, a variety of studies suggested a significant association between social media use and adolescents' psychological distress, such as depressive and anxiety symptoms (35) and the time spent on social media (36–39).

Social media use refers to engaging in communication, information and data sharing, and entertainment activities on online community-building sites that typically include acquaintances and strangers (40). While some of the approaches to social media described the aspects related to the use of social media networks or the frequency of social media use (41), Dessart et al. (42) conceptualized the use of social media through three dimensions, i.e., cognitive, affective, and behavioral. The *cognitive* dimension of social media use refers to the presence of attitudes, beliefs, and states associated with the behaviors of using social networks, displayed in the long term (42). Often, adolescents with a high level of cognitive involvement in social media use express solid beliefs about the importance of social media in their personal lives, constantly thinking about social media use (43). The *affective* dimension of social media use refers to the emotions and feelings people feel using social networks, both when they are using them and when they are not (42). For example, while using social media, individuals may feel satisfaction, happiness, or other emotions associated with social media features (44). However, in the absence of social media use, individuals may feel anxious and impatient, anticipating further use (45). Finally, the *behavioral* dimension of social media use refers to the actual behaviors of using social media, such as communicating with others, sharing news, information, images, and videos, and obtaining information about other people or important events through ads, advertisements, news, and social media posts (42).

Building on this multidimensional approach to the use of social media, Ni et al. (46) defined social media engagement as an individual attitude toward the relationship with social media use. Their approach suggested that social media engagement might replace measures such as the time spent on social media or the frequency with which users access social media better to understand the underlying mechanisms of adolescents' related behaviors. In addition, the authors also validated a scale in this regard, i.e., the Social Media Engagement Scale for Adolescents, SMES-A (2020), which we used for the current investigation.

## Adolescents and social media use effects

However, the impact of social media's use on adolescents' well-being has yet to be made clear since previous studies suggested ambivalent results regarding the valence of the impact of social media on adolescent well-being. For example, Beyens et al. (47) suggested that 44% of the adolescents in their investigated sample did not present significant changes in their well-being following the use of social media. Also, 10% of participants had significantly worse well-being, while 46% had significantly increased well-being. A subsequent longitudinal study (48) observed a similar pattern (although the positive impact of social media use was lower).

Among the reasons for using social media and its benefits, adolescents usually report the need to communicate with friends and other people in their social circle, share images and obtain information related to the activities of people they know (49). Swirsky et al. (50) also reported positive and significant associations between the frequency of social media use and prosocial support. However, more recent studies highlighted that social media presence is not critical for the self's construction, but for the feedback received for the content adolescents post (51).

Excessive social media use seems to be associated with depressive symptoms and low self-esteem [e.g., (52)], and depressive symptoms were identified as positive predictors of social media use and increased frequency of social media use in both early and late adolescence (53). In the study coordinated by Sampasa-Kanyinga and Lewis (54), social media use among adolescents was associated with higher levels of psychological distress and suicidal ideation. Also, adolescents who reported low psychological support levels spent more time on social media networks. Furthermore, Haand and Shuwang (55) reported that depressive symptoms significantly and positively predicted participants' social media addiction. However, it is important to note that such studies have an associative nature, which makes it difficult to establish causal relationships between the investigated variables. For example, depressive symptoms can be interpreted as both a cause and an effect of social media use. On the one hand, it can be assumed that the frequent use of social media networks can contribute to the emergence of depressive symptoms. On the other hand, teenagers might use the Internet as a coping mechanism, with already existing depressive symptoms causing them to use social media networks more frequently.

Sela et al. (56) suggested depressive symptoms as a predictor of excessive Internet use and time spent online through the mediating variable of fear of missing social opportunities (i.e., fear of missing out). Furthermore, the meta-analysis conducted by McCrae et al. (57) suggested significant associations between depressive symptoms and social media use. Finally, in the meta-analysis of Huang (58), excessive social media was negatively associated with participants' well-being and self-esteem and positively related to loneliness and depressive symptoms, regardless of participants' age.

### The bright side of social media use

A limited number of previous studies suggested the positive role of social media in coping with negative psychological states. For example, Cauberghe et al. (59) suggested that teenagers were more likely to use social media platforms to adapt to the changes brought by the COVID-19 pandemic rather than for communication purposes. Furthermore, the authors reported results suggested that coping through social media use may diminish the negative impact of anxiety on participants' happiness. Again, Ostic et al. (60) suggested that social capital (i.e., aspects of human social interactions, including communication networks, that allow individuals to collaborate to pursue objectives) might mediate the positive relationship between social media use and psychological well-being.

Also, a limited number of studies investigated the potential gender differences in social media use. For example, Luijten et al. (61) suggested that a higher level of social media use was reported among girls. It was also observed that girls who used social media more frequently had a lower state of well-being, which was not observed in the case of boys. Additionally, Li and Ni (62) suggested that boys and girls might express different patterns in the cognitive, affective, and behavioral use of social media networks, meaning that personality factors would explain to a greater extent the use of social media by boys (i.e., as they grow, they might gain an increased interest in forming social interactions and bonds through social media).

### Depressive symptoms

Depressive symptoms, both in their moderate forms and in situations where they can lead to the diagnosis of depressive disorders, have a considerable prevalence among the world population, including adolescents (63). For example, Shorey et al. (64) reported a global prevalence of ~34% of depressive symptoms among adolescents, 12% meeting the criteria for diagnosing depressive disorders. Their results also suggested that the prevalence of depressive symptoms and disorders seems higher among females, a pattern also found in other previous studies [e.g., (65)].

Recent studies suggested positive associations between pathologic use of the Internet and its features and depressive symptoms. In a meta-analysis, Lozano-Blasco and Cortés-Pascual (66) reported a moderate and positive relationship between pathologic internet use and depression. This effect was stronger among male participants and did not differ across cultures. Similarly, in a recent literature review, Vidal et al. (67) reported that higher frequencies of social media use were associated with higher levels of depression and suicidal ideation. The authors also highlighted the possibility of a dual relationship between depression and social media use. For example, the authors suggested that adolescents with depressive symptoms might predict higher levels of social media use in the long term and actively engage in social media use through posts and image sharing. Previous studies suggested potential reasons for these results. For example, Elmquist and McLaughlin (68) postulated that social media, despite its potentially harmful effects, can offer opportunities for coping with mental health issues (e.g., seeking advice or support from other individuals, sentiments of belonging to a group, or the possibility to access mental health resources while remaining anonymous).

Depressive disorders during adolescence are associated with later depressive episodes in adulthood, school dropout, unemployment during adulthood, and subsequent difficulties in social and professional adaptation and integration (69). Also, some previous studies suggest weak or moderate associations between adolescent depressive symptoms and social media use. For example, in a meta-analysis by Ivie et al. (36), which included 11 studies that recruited participants between the ages of 11 and 18, social media use had weak, positive, and significant associations with depressive symptoms.

## The present study

Studies to date have provided divergent results regarding the impact of social media on adolescent mental health and well-being. For example, some studies suggested a positive effect associated with higher well-being and life satisfaction. In contrast, others showed a negative effect, associated with a higher level of anxiety and depressive symptoms, insomnia, and difficulties in social integration (40). Also, some studies [e.g., (47)] have reported ambivalent effects of social media use, suggesting that a considerable number of adolescents are not significantly affected by social media use, while two groups at the extremes are affected positively or negatively. Therefore, further studies, such as the present one, are needed to understand the impact of social media use on adolescent mental health.

At the same time, our study also aimed to understand better the relationships between mental health issues and adolescents' use of social media networks. For example, previous studies suggested that social media use could contribute to depressive symptoms through exposure to adverse factors, or well-being, through engaging in enjoyable socializing and entertainment activities (49). Alternatively, depressive symptoms may lead adolescents to use social media more as a coping mechanism to cope with the adverse effects of depression by seeking social support and approval from others.

Furthermore, the results of the previous literature documenting depressive symptoms and social media use nuance in the perspective according to which the well-being of adolescents does not represent a simple absence of depressive symptoms or other mental disorders, being instead the actual presence of positive aspects, such as optimism, the formation of social connections, commitment in activities, perseverance, and happiness of teenagers (12). Furthermore, these aspects seem also to be associated with positive consequences for adolescents, such as higher school performance, better integration into the educational and social environment and reduced depressive and anxious symptoms (12, 70). However, all these studies highlight the need for further research to provide a more comprehensive view of these links, and our study answers this call.

Also, although the negative relationship between well-being and depressive symptoms is robust and intuitive, it is important to understand the potential effect of other variables, which might influence various aspects of the two factors differently. For example, some previous studies (48) suggested that external factors, such as the use of social networks, could have ambivalent effects on the well-being of adolescents. Also, in addition to some weak direct associations, social media use can significantly impact adolescent sleep quality. Consequently, lack of sleep was associated with a significantly higher presence of depressive symptoms among adolescents (71).

Given all these previous findings, the present study aims to add to the current literature regarding the relationships between these variables, especially accounting for the divergent results describing the effects of different magnitudes of the use of social media on the well-being of adolescents and young people in general. More importantly, our study addresses these issues in a highly challenging context, i.e., the COVID-19 pandemic, when Romania was heading to the peak of the deadliest COVID-19 pandemic wave.

Our study was built on two primary objectives: (1) To explore the potential associations between the use of social media at cognitive, affective, and behavioral levels and depressive symptoms and the well-being of adolescents during the COVID-19 pandemic; (2) To investigate the potential indirect effect of social media use on the relationship between depressive symptoms and adolescent well-being, during the COVID-19 pandemic.

Considering previous studies suggesting depressive symptoms as a potential predictor of social media use (53) and social media use as a potential predictor of well-being, we assumed that (*H1*) Social media use (i.e., the cognitive, affective, and behavioral dimensions) would mediate the relationship between depressive symptoms and participants' well-being. A higher level of depressive symptoms would be associated with a higher level of social media use, which would be related to a higher level of well-being. Also, considering the possible gender differences in the use of social media (61), we assumed *(H2)* that there would be significant differences between male and female adolescents regarding their social media use, with female participants scoring higher than male participants.

## Method

### Participants and procedure

Our convenient research sample was formed of 258 adolescents aged 11–16 years (*M* = 13.38, *SD* = 0.93), out of which 147 were males (57%). The participants were recruited from three educational institutions in a northeastern town in Romania. The data were collected between September 23, 2021, and October 7, 2021, as a part of a project aiming to explore adolescents' well-being. During that period, the schools were reopened, and the scales were filled in person (paper and pencil procedure). However, not all schools were open. Even in the schools where our participants were students, online teaching was alternated with offline classes in a cyclical system to avoid spreading the virus. Thus, we collected our data during the time when the students were physically present in schools.

The research was conducted following the Helsinki Declaration's ethical criteria and the ethical research requirements approved by the institutional board of the authors' institution. Permission was also received from the schools' principals, as well as the parents of the participants, who were informed of the study's goal and methodology. After receiving these approvals, we began the data collection process. Participants who agreed to participate voluntarily were told of the contents of the research, the tasks they were required to do (i.e., to answer the scales' items), the possibility to withdraw from the study at any time, and the confidentiality and anonymity of their answers, which would only be used solely for the present research. The participants gave informed consent to take part in this research. Furthermore, the participants were informed that none of their answers or participation would impact their academic performance or other academic-related variables. The average completion time was ~15 min.

## Measures

Before using the instruments, we followed the cross-cultural adaptation methods indicated by the International Test Commission (72) and related recommendations [e.g., (73, 74)]. Using the experience of two independent translators, we first translated the original instrument into the target language, i.e., from English to Romanian. Using the input of a third independent translator, we compared the two translated versions and evaluated the potential ambiguities and discrepancies of words, sentences, and meanings. There were no acknowledged significant inconsistencies, and the consensus reached permitted the tentative initial version of the translated scales. We next conducted a blind back-translation of the initial preliminary translation of the instruments, followed by a comparison of the two back-translated scales., which resulted in the final versions of the instruments.

### Social media use

We used the Social Media Engagement Scale for Adolescents (46) to measure adolescents' social media use. The scale consists of 11 items, each item presenting a statement regarding certain aspects of the use of social media networks. Participants provided their answers on a Likert scale ranging from 1 (“not true at all”) to 5 (“very true”). The scale comprises three dimensions. The first dimension, the *behavioral* use of social media networks, includes four items (e.g., “Using social media is my daily habit”), with a Cronbach's alpha of 0.79. The second dimension, the *cognitive* use of social media networks, includes three items related to participants' cognitions related to the use of social networks (e.g., “Support and encouragement of others on social media are very important for me”), with a Cronbach's alpha of 0.84. The third dimension, the *affective* use of social media networks, includes four items related to possible emotional reactions related to social media use (e.g., “I feel anxious when I can't use social media”), Cronbach's alpha of 0.84. High scores indicated a higher level of social media usage at that level.

### Well-being

We used the 20-item EPOCH Measure of Adolescent Well-Being scale (12). Each item presents a statement regarding various aspects related to the proper functioning and well-being of the participants. Participants indicated to what extent they agreed with each statement on a Likert scale ranging from 1 (“not true at all”) to 5 (“very true”). The scale comprises five dimensions, i.e., *Engagement* (e.g., “When I do an activity, I enjoy it so much that I lose track of time.”), *perseverance* (e.g., “I finish whatever I start”), *Optimism* (e.g., “I think good things are going to happen to me.”), *Connection* (e.g., “When I have a problem, I have someone who will be there for me.”), and *happiness* (e.g., “I have a lot of fun.”). High scores indicated a higher level of well-being. Cronbach's alpha for the overall scale was 0.91.

### Depression

We used the 11-item Adolescent Depression Rating Scale (75) to measure participants' depressive symptoms. Participants provided their answers on a Likert scale ranging from 1 (“not true at all”) to 5 (“very true”), and higher scores indicated higher symptoms. Example items include “I feel overwhelmed by sadness and listlessness” and “I feel downhearted and discouraged”. Cronbach's alpha was 0.90.

Finally, a demographic scale assessed participants' gender and age.

## Overview of the statistical analysis

We conducted preliminary analyses (using the IBM SPSS 26 statistical software) to assess whether participants' age relates to the primary variables of interest. Also, zero-order correlations among the main study variables were computed. Finally, we investigated the mediating effect of each social media use dimension on the relationship between depressive symptoms and adolescents' well-being.

## Results

Descriptive statistics of the study variables (mean, standard deviation, minimum and maximum values, Skewness and Kurtosis coefficients, Alpha Cronbach's coefficients) are reported in Table 1.

**Table 1:** Descriptive statistics for the primary variables (*N* = 258).

	***M***	**SD**	**Min**	**Max**	**Skewness (SD)**	**Kurtosis (SD)**	**α**
Well-being	18.75	3.31	5	25	0.01 (0.15)	−0.23 (0.30)	0.91
Social media use (overall)	34.52	9.80	11	55	−0.29 (0.15)	−0.02 (0.30)	0.90
Affective (social media use)	14.63	4.12	4	20	−0.58 (0.15)	−0.24 (0.30)	0.84
Behavioral (social media use)	9.02	3.10	3	15	−0.10 (0.15)	−0.37 (0.30)	0.84
Cognitive (social media use)	10.86	4.22	4	20	0.21 (0.15)	−0.53 (0.30)	0.79
Depressive symptoms	23.77	9.10	10	50	0.56 (0.15)	−0.22 (0.30)	0.90

### Associations between the variables

The results of Pearson correlations are reported in Table 2. Adolescents' well-being was positively associated with overall social media use and the behavioral and affective dimensions. At the same time, adolescents' well-being was negatively correlated with depressive symptoms and age: older participants reported a lower level of well-being. The behavioral and affective dimensions of social media use were also positively associated with depressive symptoms, while the affective dimension of social media use and depressive symptoms were positively correlated with participants' age.

**Table 2:** Zero-order correlations between the main variables (*N* = 258).

	**M**	**SD**	**1**	**2**	**3**	**4**	**5**	**6**
1. Well-being	18.75	3.31	–					
2. Social media use (overall)	34.52	9.80	0.22^**^	–				
3. Affective (social media use)	10.86	4.22	0.11	0.87^**^	–			
4. Behavioral (social media use)	9.02	3.10	0.34^**^	0.85^**^	0.69^**^	–		
5. Cognitive (social media use	14.63	4.12	0.16^**^	0.83^**^	0.53^**^	0.57^**^	–	
6. Depressive symptoms	23.77	9.10	−0.32^**^	0.28^**^	0.34^**^	0.12	0.24^**^	–
7. Age	13.38	0.93	−0.21^**^	0.15^*^	0.08	0.03	0.25^**^	0.12^*^

* p < 0.05;

** p < 0.01.

### Gender differences

Next, to observe potential differences between male and female participants on study variables, we applied independent samples *t-*tests (see Table 3). Results of independent sample *t-*tests suggest no significant differences between male and female participants on well-being, overall social media use score, behavioral or cognitive dimensions of social media use, or depressive symptoms. The only significant differences were observed in the affective dimension of social media use. More specifically, female participants reported significantly higher levels of social media use on the affective dimension than male participants.

**Table 3:** Independent T-test results (*N* = 258).

	**Self-reported gender**		
	**Male (*N*=147)**	**Female (*N*=111)**	***t***
	***M (SD)***	***M (SD)***	
Well-being	18.72 (3.26)	18.80 (3.39)	−0.196
Social media use (overall)	33.54 (10.06)	35.82 (9.33)	−1.864
Affective (social media use)	13.90 (4.07)	15.60 (3.99)	−3.342^***^
Behavioral (social media use)	8.93 (3.19)	9.14 (2.98)	−0.519
Cognitive (social media use	10.69 (4.25)	11.08 (4.19)	−0.723
Depressive symptoms	22.99 (8.71)	24.80 (9.53)	−1.583

*** p < 0.001.

### Mediation analyses

Based on these findings, we further performed three mediation analyzes, using depressive symptoms as an independent variable, well-being as a dependent variable, and each dimension (cognitive, behavioral, and affective) of social media use as mediating variables. We used Hayes' (76) macro Process (Model 4; 95% confidence interval (CI); 5000 bootstrapped samples).

*a. The mediating role of the cognitive dimension of social media use on the relationship between depressive symptoms and participants' well-being*.

**Figure 1:**
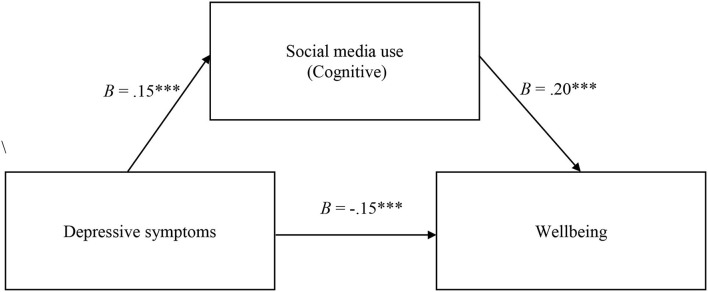
Cognitive social media use partially mediates the relation between depressive symptoms and wellbeing. The symbol “***” indicates a *p*-value lower than 0.001.

The total effect of depressive symptoms on participants' well-being was significant (*B* = −0.11, *SE* = 0.02, *p* <0.001, 95% CI [−0.15; −0.07]), a higher level of depressive symptoms being associated with a lower level of well-being. The direct effect of depressive symptoms on well-being was significant as well (*B* = −0.15, *SE* = 0.02, *p* < 0.001, 95% CI [−0.19; −0.10]). The effect of depressive symptoms on the cognitive dimension was significant as well (*B* = 0.15, *SE* = 0.02, *p* <0.001, 95% CI [0.10; 0.21]). The effect of cognitive dimension on well-being was significant (*B* = 0.21, *SE* = 0.04, *p* <0.001, 95% CI [0.11; 0.30]). The indirect effect of depressive symptoms on well-being through the cognitive dimension of social media use was significant (*B* = 0.03, *SE* = 0.01, 95% CI [0.01; 0.05]), with higher depressive symptoms being associated with a higher level of cognitive involvement in social media use. At the same time, a higher level of social media use was associated with a higher level of well-being. The results indicated a partial mediating effect of the cognitive dimension of social media use on the relationship between depressive symptoms and well-being (see Figure 1).

*b. The mediating role of the behavioral dimension of social media use on the relationship between depressive symptoms and participants' well-being*.

**Figure 2:**
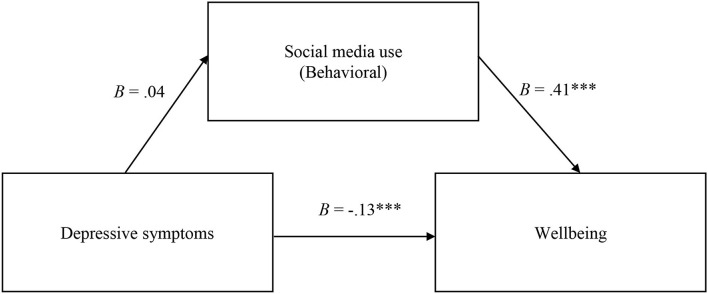
No significant mediation effect of behavioral social media use on the relation between depressive symptoms and wellbeing. The symbol “***” indicates a *p*-value lower than 0.001.

The results of the second mediation model are presented in Figure 2. They indicate that the total effect of depressive symptoms on well-being was significant (*B* = −0.11, *SE*= 0.02, *p* < 0.001, 95% CI [−0.15; −0.07]), a higher level of depressive symptoms being associated with a lower level of well-being. The direct effect of depressive symptoms on well-being was significant as well (*B* = −0.13, *SE*= 0.02, *p* < 0.001, 95% CI [−0.17; −0.09]). The effect of depressive symptoms on the behavioral dimension of social media use was not significant (*B* = 0.04, *SE* = 0.02, *p* > 0.05, 95% CI [0.00; 0.08]). The effect of the behavioral dimension of social media use on well-being was significant (*B* = 0.41, *SE* = 0.05, *p* < 0.001, 95% CI [0.29; 0.52]), with a higher level of behavioral involvement being associated with a higher level of well-being. The indirect effect of depressive symptoms on well-being, through the cognitive dimension, was not significant (*B* = 0.01, *SE* = 0.01, 95% CI [−0.00; 0.03]). Thus, the results indicate a non-significant mediating effect of the behavioral dimension of social media use on the relationship between depressive symptoms and well-being.

*c. The mediating role of the affective dimension of social media use on the relationship between depressive symptoms and participants' well-being*.

**Figure 3:**
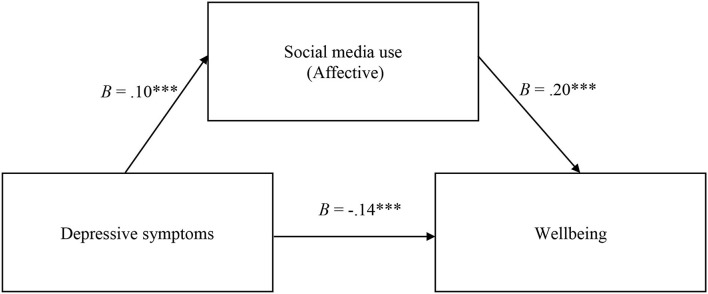
Affective social media use partially mediates the relation between depressive symptoms and wellbeing. The symbol “***” indicates a *p*-value lower than 0.001.

The results of the third mediation model are presented in Figure 3. These indicate that the total effect of depressive symptoms on well-being was significant (*B* = −0.11, *SE* = 0.02, *p* < 0.001, 95% CI [−0.15; −0.07]), a higher level of depressive symptoms being associated with a lower level of well-being. The direct effect of depressive symptoms on well-being was significant (*B* = −0.14, *SE*= 0.02, *p* < 0.001, 95% CI [−0.18; −0.09]). The effect of depressive symptoms on the affective dimension of social media use was significant (*B* = 0.10, *SE* = 0.02, *p* < 0.001, 95% CI [0.05; 0.16]). The effect of the affective dimension of social media use on well-being was significant (*B* = 0.21, *SE* = 0.04, *p* < 0.001, 95% CI [0.12; 0.31]). The indirect effect of depressive symptoms on well-being through the affective dimension of social media use was significant (*B* = 0.02, *SE* = 0.00, 95% CI [0.009; 0.04]). A higher level of depressive symptoms was associated with a higher level of the affective dimension of social media use, which in turn was associated with a higher level of well-being. The results indicate a partial mediating effect of the affective dimension of social media use on the relationship between depressive symptoms and well-being. When gender was introduced as a covariate, we observed a partial mediating effect (direct effect: *B* = −0.14, 95% CI [−18; −0.09], indirect effect: *B* = 0.02, 95% CI [0.007; 0.04]).

## Discussion

Across all mediation analyses, our results suggested that social media use positively predicted adolescents' well-being. These results aligned with previous studies (48), suggesting the ambivalent effects of social media use on well-being, with the present study providing results in favor of the positive impact of social media use. Taking into account the particularities of the instrument used to measure social media use, adolescent well-being was predicted not only by actual social media use behaviors but also by cognitions related to the expectation of receiving gratification on social media and by the intense affective states related to the desire to use social media.

Consequently, the relationship between these variables (especially in the COVID-19 pandemic context) might be interpreted in a positive key, improving the well-being of adolescents by using the facilities offered by social media (communication with close people, building and maintaining social relationships, sharing and retrieving information and data), but as well in a less positive key, using maladaptive coping mechanisms, participants associated the use of social media with pleasant feelings, high expectations of receiving gratification and distraction from problems. Previous findings support this interpretation. For example, Matthes et al. (77) suggested that using social media sites such as YouTube, WhatsApp and Snapchat might contribute to perceptions of information overload, leading to more depressive symptoms. On the other hand, Cauberghe et al. (59) suggested that active coping through social media use mediated the relationship between anxiety and happiness, with higher levels of anxiety being associated with higher levels of active coping through social media use, which in turn led to higher levels of happiness.

The cognitive dimension of social media use partially mediated the relationship between depressive symptoms and well-being. Depression had a direct negative effect on well-being, but it positively affected the cognitive dimension of social media use, which positively impacted well-being. These specific results suggested that adolescents with high levels of depressive symptoms are more likely to capitalize on social media use and to have expectations related to receiving approval from others in the context of social media use. Consequently, building this social media image is associated with a higher level of well-being. In addition, these findings also suggested that adolescents may use cognitive engagement in social media use as a coping mechanism.

In line with other similar studies [e.g., (78)], our results might sustain the idea that in the case of youth who presented depressive symptoms before the pandemic, COVID-19 might have had even a more substantial impact on their well-being due to isolation, less direct peer interaction and overall predictability. In such a context, relying on the digital universe might have been enforced both as a solution to continue education and as the sole means to keep in contact with others. Thus, our results might signal a potential path to the positive role of social media capital in building their identity if facing depressive symptoms that hinder their direct participation, also after the pandemic.

On the other hand, the behavioral dimension of social media use did not mediate the relationship between depressive symptoms and well-being. Thus, depressive symptoms did not significantly impact adolescents' actual use of different social media functions, as defined by Ni et al. (46). However, the behavioral dimension of social media use positively predicted adolescent well-being, having the most substantial effect on the dependent variable among all mediating variables. These results suggested that actual use of social media may improve adolescent well-being, but depressive symptoms do not cause them to change their frequency of use or time spent on social media. Alternatively, these results could be explained by depressive symptoms, with adolescents high in depressive symptoms being apathetic, thus having little interest in engaging in activities that would ordinarily interest them, be it that we refer to direct or virtual activities. A secondary line of explanation can be linked to the period of the data collection (fall of 2021, returning to school after a significant period of online schooling); at this point of the pandemic, we can talk of a potential decrease in the use of social media as a consequence of boardroom, fatigue associated to being only online and an overall reduction of interest in social media, as direct exposure to peers was again possible [similar results reported by Longest and Kang (79)]. At the same time, the lack of significant correlations between depressive symptoms and the behavioral dimension of social media use may suggest that the association between the two variables could be weak, making it difficult to establish a causal relationship between them. In this regard, previous studies have suggested weak or moderate associations of depression with social media use, with many identifying only a small effect in the relationship between the two variables (58).

Next, the affective dimension of social media use partially mediated the relationship between depressive symptoms and well-being. Depression had a negative effect on well-being, but it had a positive effect on the affective dimension of social media use, which positively impacted adolescent well-being. These results suggest that depressive symptoms may lead adolescents to compare physical and online social interactions and conclude that social media interactions are more enjoyable than traditional ones. Consequently, these attitudes could lead adolescents to spend more time on social networks, contributing to increased well-being. Similar to the pattern observed in the relationship between depression, the cognitive dimension of social media use, and well-being, these results could suggest that adolescents use social networks as a maladaptive coping mechanism to deal with depressive symptoms.

The present results indicated significant gender differences only at the affective dimension of social media use level, only partially confirming the second hypothesis. The results suggest that female participants show a higher level of affective involvement in the use of social media than boys. Furthermore, these results suggested a greater preference for teenage girls to socialize online over traditional socializing. Given the lack of significant differences between the two groups on all other study variables, these differences cannot be adequately explained by variance in variables such as the prevalence of depressive symptoms or different dimensions of social media use. Also, although the results related to gender differences in social media use are in line with previous studies, these results are contradictory to some previous studies where girls reported a higher level of social media use, which was associated with a lower level of well-being (61), in this study no gender differences in well-being were observed. To explain these divergences, it is important to note that social media use refers to socializing through sites built primarily to serve this purpose (such as Facebook, Twitter, and Instagram). However, virtual socializing can also occur in the context of multiplayer or online-only video games, with previous research suggesting a much higher prevalence of video game socializing among boys, with girls preferring to socialize through social media networks (80). Therefore, in further studies, it would be necessary to adopt a broader conceptualization of socialization in the online context, which includes more modalities in addition to using social networks.

The results of this study suggested the potential role of social media use by adolescents as a coping mechanism to deal with depressive symptoms and improve their well-being. At the same time, the study's results suggested that different dimensions of social media use may play varied roles in the relationship between depressive symptoms and well-being, the behavioral dimension of social media use not being influenced in the same way as the cognitive or affective dimension.

Several limitations of the present study need further attention. First, we used a convenience sample, limiting the possibility of generalizing these results to the adolescent population. Further studies consider using larger and more representative samples. A second limitation is related to the self-reported scales that we used, which may have led participants to provide socially desirable responses. At the same time, they require introspection and subjective evaluations. Therefore, further studies could use more objective methods, such as experimental approaches to observe the relationship between the investigated variables.

Further studies may consider the introduction of new variables relevant to the investigation of adolescent well-being and depressive symptoms, such as fear of missing out (56), and may use instruments alternatives to gain a better understanding of the relationship between social media use and adolescent depressive symptoms and well-being. Also, for a better understanding of the impact of online socialization on adolescents' well-being and depressive symptoms, it would be recommended to use more comprehensive conceptualizations that include, in addition to social media networks, other platforms that facilitate online socializing, such as video games, blogs, problem-solving sites. Also, the EPOCH approach presents specific limits [e.g., the cultural impact for each research setting (81)], which future studies might address. Finally, we did not specifically assess the impact of the COVID-19 pandemic nor the related experiences, which might have accounted for some variations in our results.

## Conclusion

Previous studies examining the impact of the actual use of social media networks on depression and the well-being of adolescents paid little attention to other factors associated with using social networks (such as cognitive and affective involvement), especially during the COVID-19 pandemic. The present study nuanced the perspective on these variables, suggesting that depressive symptoms may not be directly associated with actual behaviors of regularly using social media but rather with some maladaptive cognitive and affective processes related to social media use. Also, this study contributes to the understanding of gender differences in social media use, suggesting a tendency for teenage girls to show maladaptive affective reactions related to social media use at a significantly higher level than male teenagers. However, further studies are needed to test the relevance of these conclusions.

## Data availability statement

The original contributions presented in the study are included in the article/supplementary files, further inquiries can be directed to the corresponding author.

## Ethics statement

The studies involving human participants were reviewed and approved by FPSE Iasi. Written informed consent to participate in this study was provided by the participants' legal guardian/next of kin.

## Author contributions

All authors listed have made a substantial, direct, and intellectual contribution to the work and approved it for publication.
